# Robust generation of human-chambered cardiac organoids from pluripotent stem cells for improved modelling of cardiovascular diseases

**DOI:** 10.1186/s13287-022-03215-1

**Published:** 2022-12-21

**Authors:** Beatrice Xuan Ho, Jeremy Kah Sheng Pang, Ying Chen, Yuin-Han Loh, Omer An, Henry He Yang, Veerabrahma Pratap Seshachalam, Judice L. Y. Koh, Woon-Khiong Chan, Shi Yan Ng, Boon Seng Soh

**Affiliations:** 1 Disease Modelling and Therapeutics Laboratory, Institute of Molecular and Cell Biology, Agency for Science, Technology and Research (A*STAR), 61 Biopolis Drive Proteos, Singapore, 138673 Singapore; 2 Department of Biological Sciences, National University of Singapore, 16 Science Drive 4, Singapore, 117543 Singapore; 3 Integrative Sciences and Engineering Programme, National University of Singapore, 21 Lower Kent Ridge Road, Singapore, 119077 Singapore; 4 Epigenetics and Cell Fates Laboratory, Institute of Molecular and Cell Biology, Agency for Science, Technology and Research (A*STAR), 61 Biopolis Drive Proteos, Singapore, 138673 Singapore; 5 Cancer Science Institute of Singapore, National University of Singapore, 14 Medical Drive, Singapore, 117599 Singapore; 6 Computational Phenomics Group, Experimental Drug Development Centre (EDDC), Agency for Science, Technology and Research (A*STAR), Singapore, 138670 Singapore; 7 Neurotherapeutics Laboratory, Institute of Molecular and Cell Biology, Agency for Science, Technology and Research (A*STAR), 61 Biopolis Drive Proteos, Singapore, 138673 Singapore; 8 Department of Physiology, Yong Loo Lin School of Medicine, National University of Singapore, 10 Medical Drive, Singapore, 117456 Singapore; 9 National Neuroscience Institute, 11 Jalan Tan Tock Seng, Singapore, 308433 Singapore

**Keywords:** Cardiac progenitor, Chambered cardiac organoid, Disease modelling, Human pluripotent stem cells, Single-cell RNA sequencing

## Abstract

**Background:**

Tissue organoids generated from human pluripotent stem cells are valuable tools for disease modelling and to understand developmental processes. While recent progress in human cardiac organoids revealed the ability of these stem cell-derived organoids to self-organize and intrinsically formed chamber-like structure containing a central cavity, it remained unclear the processes involved that enabled such chamber formation.

**Methods:**

Chambered cardiac organoids (CCOs) differentiated from human embryonic stem cells (H7) were generated by modulation of Wnt/ß-catenin signalling under fully defined conditions, and several growth factors essential for cardiac progenitor expansion. Transcriptomic profiling of day 8, day 14 and day 21 CCOs was performed by quantitative PCR and single-cell RNA sequencing. Endothelin-1 (EDN1) known to induce oxidative stress in cardiomyocytes was used to induce cardiac hypertrophy in CCOs in vitro. Functional characterization of cardiomyocyte contractile machinery was performed by immunofluorescence staining and analysis of brightfield and fluorescent video recordings. Quantitative PCR values between groups were compared using two-tailed Student’s *t* tests. Cardiac organoid parameters comparison between groups was performed using two-tailed Mann–Whitney *U* test when sample size is small; otherwise, Welch’s *t* test was used. Comparison of calcium kinetics parameters derived from the fluorescent data was performed using two-tailed Student’s *t* tests.

**Results:**

Importantly, we demonstrated that a threshold number of cardiac progenitor was essential to line the circumference of the inner cavity to ensure proper formation of a chamber within the organoid. Single-cell RNA sequencing revealed improved maturation over a time course, as evidenced from increased mRNA expression of cardiomyocyte maturation genes, ion channel genes and a metabolic shift from glycolysis to fatty acid ß-oxidation. Functionally, CCOs recapitulated clinical cardiac hypertrophy by exhibiting thickened chamber walls, reduced fractional shortening, and increased myofibrillar disarray upon treatment with EDN1. Furthermore, electrophysiological assessment of calcium transients displayed tachyarrhythmic phenotype observed as a consequence of rapid depolarization occurring prior to a complete repolarization.

**Conclusions:**

Our findings shed novel insights into the role of progenitors in CCO formation and pave the way for the robust generation of cardiac organoids, as a platform for future applications in disease modelling and drug screening in vitro.

**Supplementary Information:**

The online version contains supplementary material available at 10.1186/s13287-022-03215-1.

## Introduction

Deriving tissue types from human pluripotent stem cells (hPSCs) for use in disease modelling is one cornerstone for biomedical research. Traditionally, hPSC-derived cell types are cultured in 2D for routine disease phenotyping and drug screening [1]. However, these models representing various tissues and organs inherently lack the structurally complex elements of their in vivo counterparts. Therefore, there is a push to develop 3D cultures that recapitulate the microenvironment, cell–cell interactions, architectural and geometric features of tissues in vivo [2–7].

In the context of cardiovascular diseases, defects in heart structure, contraction rhythms and conduction flow similarly are lacking proper representation in 2D cultures. Thus, these cardiac parameters are modelled using 3D cardiac model alternatives primarily generated using scaffold systems. However, such models typically lack the cellular heterogeneity present in the heart, are unable to recapitulate in vivo cardiogenesis and are thus inadequate disease models of the adult heart [8, 9]. Only recently was it demonstrated by Lee et al*.* that mouse cardiac organoids mimicking developmental processes in a spatial and temporal manner could be intrinsically formed [10, 11]. Thereafter, the first 3D human cardiac organoids were intrinsically generated with an inner structure that resembled a cardiac chamber [12, 13]. Drakhlis et al*.* utilized cardiac organoids termed heart-forming organoids (HFO) to highlight the necessity of Wnt pathway modulation to enable the self-organization of embryo-like tissue patterns in vitro. While these findings advanced the field of generating 3D models, the exact cellular and molecular details on how Wnt signalling enabled the self-organization are uncertain. Notably, methodology for utilizing the chamber-like structures for disease modelling has not been explored.

Herein, we sought to bridge the gap in the field of 3D cardiovascular disease modelling, by first differentiating and characterizing chambered cardiac organoids. Leveraging on findings established by Soh et al*.*, human cardiovascular progenitors (CVPs) that were expanded and maintained in vitro in the presence of Wnt3A and EDN-1, possessed the intrinsic ability to self-organize, forming vasculature and cardiac structures when xenografted in vivo [14]. We reasoned that Wnt modulation was essential for the presence or enrichment of CVPs to self-organization into chamber-like structures within the cardiac organoid. By titrating various levels of CVPs used for organogenesis, we further demonstrated the critical role of CVPs for robust generation of 3D chambered cardiac organoids (CCO) in vitro. The multi-lineage CCOs were characterized using single-cell RNA sequencing and were shown to exhibit improved maturation over a time course, thus making them suitable for disease modelling. Lastly, the CCOs were utilized to model cardiac hypertrophy, typically characterized as a consequence of increased CM size and change in other heart muscle components, such as the extracellular matrix [15, 16]. While 2D PSC-derived cardiomyocytes provide a platform for modelling cardiac hypertrophy, these studies fall short in terms of global gene expression profiles, the differentiation of various cell types, and more importantly, compartmentalization of a chambered organoid structure to model diseases associated with the thickening of the heart wall. In this study, we outline the methodology to derive structural and functional parameters that take advantage of the presence of a cardiac chamber, such as chamber wall thickness and fractional shortening. Other functional abnormalities such as contraction rhythm, electrophysiology and sarcomeric alignments were also consistent with the clinical observations of patients with cardiac hypertrophy. Collectively, these results provide an understanding for the generation of chambered cardiac organoids to be utilized for in vitro disease modelling of a human heart.

## Methods

### Pluripotent stem cell lines and organoid culture

The human H7 hES cell line (WiCell; hPSCReg ID: WAe007-A) was cultured in feeder-free conditions on culture plates coated with Matrigel® matrix (Corning, U.S.A.) and maintained in StemMACS™ iPS-Brew XF medium (Miltenyi Biotec). Culture medium was changed daily. After 80–90% confluency was reached, hES were passaged using ReLeSR (STEMCELL Technologies).

At 90–95% confluency, differentiation was started using RPMI-1640 Medium (1x) (Hyclone) with B-27 Minus Insulin 50 × supplement (Gibco, U.S.A.) and 1% Pen/Strep (Gibco, U.S.A) with the addition of 12 µM CHIR99021, a GSK inhibitor (Miltenyi Biotec), as described by Lian et al*.,* 2013 [17]. After 24 h in culture, CHIR99021 was removed and just the basal RPMI/B27 minus insulin was used for 2 days. On day 3, 5 µM IWP-2 (Miltenyi Biotec) was added into the basal media. On day 5, IWP-2 was removed, and basal RPMI/B27 minus insulin was used for 2 days.

To generate the organoids on day 7, the cells were dissociated into single cells using StemPro-Accutase® (Gibco, U.S.A.). The cells were resuspended in CGM media, and 500k cells were seeded into each well of a 96 well, ultra-low attachment round bottom plate (CoSTAR, 2022-03-14). The plate was subsequently spun down at 1200 rpm for 5 min, and the supernatant was removed from each well. With 2 × Matrigel® matrix (Corning, U.S.A.) diluted in DMEM/F12 (Gibco, U.S.A.), 20 µl of Matrigel was added into each well, followed by a quick spin at 1200 rpm for 5 min. The 96-well plate was incubated at 37 °C under humidified atmosphere of 5% CO_2_ for an hour. After one hour, 100 µl of CGM media (B27 + supplement, 17.5 ng/ml Thrombin, 3 ng/ml Cardiotrophin-1, 2 ng/ml EGF, 5 ng/ml FGF-2, 20 ng/ml Wnt-3A, 20 ng/ml EDN1) was added into each well, drop-by-drop. Fresh media was changed every two days. On day 14, organoids were transferred from the 96 well plates into a spinner flask containing 50 ml of RPMI/B27 media supplemented with 1% Pen/Strep and placed on a spinner platform at 60 rpm.

### Fluorescence-activated cell sorting and flow cytometric analysis

On day 7 of the cardiomyocyte differentiation, the cells were dissociated using StemPro-Accutase®(Gibco, U.S.A.) and stained with EDNRA antibody (1:200 dilution, Invitrogen; #MA3-005) for 2 h at 37 °C in blocking buffer (1% FBS in PBS). After 2 h, the cells were washed and stained with anti-human CD172a/b (SIRPα/β) antibody conjugated antibody (1:250 dilution, Biolegend; #323808) and donkey anti-rabbit Alexa Fluor® 594 secondary antibody (1:1000 dilution, Invitrogen; #R37119) for 1 h. The cells were washed thoroughly and resuspended in FACS buffer (0.5% FBS, 1% BSA in PBS), and FACS was performed using LSR II (BD Biosciences, U.S.A.).

### Immunohistochemistry

Organoids were washed in PBS before fixing in 4% PFA and placed on a rotator in 4 °C overnight. The excess fixative was removed with three washes of PBS, and organoids were then incubated in a 15% sucrose solution in 4 °C overnight, followed by 30% sucrose in 4 °C overnight. Finally, the organoids were embedded in OCT for snap-freezing on dry ice. For immunohistochemical stainings, organoids were sectioned in slices of 10 µm thickness at − 15 to − 20 °C. Then, sections were stored in − 70 °C for long-term storage. For immunostaining, organoid sections were washed with PBS thrice and the tissue was fixed with 4% PFA for 10 min, followed by permeabilizing solution (0.1% Triton-X in PBS) for 10 min. Afterwards, sections were incubated overnight in 4 °C in blocking buffer (5% FBS in PBS) containing antibodies anti-cTnT (rabbit,1:400, Abcam, ab45932), anti-CleavedCaspase-3 (rabbit, 1:400, CST, D175) anti-KI67 (mouse, 1:800, CST, 8D5). On the next day, sections were washed thrice with PBS and incubated for 1–1.5 h at room temperature in secondary antibody in Alexa Fluor® 594-conjugated anti-rabbit antibody (1:1000), Alexa Fluor® 594-conjugated anti-mouse antibody (1:1000), Alexa Fluor® 488-conjugated anti-rabbit antibody (1:1000), Alexa Fluor® 594-conjugated anti-mouse antibody (1:1000). Stained organoid cryosections were imaged using Olympus FV1000 Inverted Confocal Microscope (10x/0.3 UPlanFL N, 20x/0.5 LUCPlanFL N, 100x/1.4 UPlanSApo Oil objective lenses), 3 × FV10-SPD confocal detector. Image resolution was captured at 840 × 840 pixels using FluoView 4.2.

### Confocal microscopy image acquisition and analysis

Sarcomeric imaging and processing were similarly performed using Olympus FV1000 Inverted Confocal Microscope and FluoView4.2. Sarcomere length and myofibril width were measured using a custom Fiji plug-in, MyofibrilJ, available from (https://imagej.net/MyofibrilJ). All measurements were based on α-actinin staining. Full details of this analysis were as published by Spletter et al. [18]. Results were recorded in Excel, and statistical analysis comparing two groups was performed by mean of a two-tailed unpaired Student’s *t* test. *P* values lower than 0.05 were considered significant.

For measurement of skewness of CZL and Z-line fraction, primary antibody anti-sarcomeric α-actinin (1:150, ab9465, Abcam) was incubated at 4 °C overnight, followed by incubation with anti-mouse Alexa Fluor® 488 and Alexa Fluor®568 conjugated phalloidin dyes for 1.5 h, and counter-stained with 4,6-diamindino-2-phenylindole (DAPI) (AAT Biorequest, U.S.A.). The coverslips were mounted on a glass slide, and imaging was performed with the Olympus Fluoview inverted confocal microscope (Olympus, U.S.A.) using oil immersion for 100 × optical magnification. A computational tool, ZlineDetection, was performed on MATLAB to determine skewness of continuous z-line (CZL) and z-line fraction as described by Morris et al. [19]. All measurements were based on α-actinin (green) and phalloidin (red) staining. Statistical analysis comparing two groups was performed by mean of a two-tailed unpaired Student’s *t* test. *P* values lower than 0.05 were considered significant.

### Magnetic bead sorting of cardiomyocyte

hES-derived cardiomyocytes and cardiac organoids were dissociated with StemPro-Accutase®(Gibco, U.S.A.); cells were blocked with a solution containing 0.5% bovine serum albumin (BSA) and 2 mM EDTA. Using human PSC-derived cardiomyocyte isolation kit (Miltenyi Biotec, Germany), cardiomyocytes were isolated using a two-step isolation protocol as per the manufacturer’s instructions.

### RNA extraction

For cultured cell samples, cells were collected and lysed in 300 µl of TRIzol reagent (Invitrogen, U.S.A.). The samples were allowed to stand for 5 min at room temperature, after which 180 µl of chloroform (Kanto Chemical, Japan) was added to allow for phase separation by centrifugation at 12,000×*g* for 15 min at 4 °C. Next, aqueous phase was transferred to a fresh tube with equal volumes of isopropanol and GlycoBlue Coprecipitant (Invitrogen, U.S.A.). The samples were incubated at room temperature for 20 min. The samples were pelleted through centrifugation at 12,000×*g* for 15 min at 4 °C. The RNA pellet was washed with 100% ethanol and air-dried before reconstituting it in nuclease-free water (Ambion, U.S.A.).

### Reverse transcription and quantitative real-time PCR

RNA samples (250–500 ng) were reverse transcribed to obtain cDNA using High-Capacity cDNA Reverse Transcription Kit (Applied Biosystems, U.S.A.). qPCR was performed using the FAST SYBR Green Mix (Applied Biosystems, U.S.A.), 0.3 μM of specific primers (Additional file 15: Table S3) and ~ 5 ng of cDNA. ΔΔC_T_-based relative quantification method was adopted for qPCR analysis using the QuantStudio 5 384-well Block Real-Time PCR system (Applied Biosystems, U.S.A.). The threshold cycle was determined to be ≥ 35. Data are presented as fold-change where CT values were normalized to *β-ACTIN*. Data presented are representative of three independent experiments with error bars indicative of the standard deviation unless otherwise stated.

### Organoid dissociation

Organoids of various ages (Day 8, Day 14, and Day 21) were dissociated using TrypLE (ThermoFisher, 12,604,013). Organoids were washed once with 1xPBS and then incubated in TrypLE solution at 37 °C for 10 to 15 min. Subsequently, organoids were dissociated into single cells by gentle pipetting using wide-bore tips. Cell solutions were then diluted with 1xPBS and passed through 40um strainer (Falcon, 352,340) to remove any undissociated cell clumps. Cells were then centrifuged down to remove TrypLE and washed once with 1xPBS (300×*g* for 5 min at 4 °C). Pellets were eventually re-suspended in 1xPBS and stored on ice.

#### 10x genomics single-cell RNA Library preparation and sequencing

Single-cell RNA sequencing was performed at Mayo Clinic, by Dr. Hu Li. HiSeq4000 instrument was used to sequence the samples (sequencing parameters: pair-end 150 bp). Cell viability and concentration were checked using an automated cell counter (BioRad). Cell stocks were diluted to around 1000 cells/μL with 1xPBS before downstream steps. Chromium™ Single Cell 3’ Library & Gel Bead Kit v2 was used to construct the library. For all library preparation, recovery of 6000 cells was aimed. Briefly, cells together with gel bead-in-emulsions (GEM)-reverse transcription reagents, and GEM beads were loaded to the 10xChip, in which individual cells are captured, lysed, reverse transcribed and barcoded. Subsequently, barcoded cDNA was amplified and used for library construction. Libraries which passed the quality control were then sent out for sequencing.

### Bioinformatics

Cell filtering, data normalization and unsupervised analysis were carried out using R library *Seurat* version 3.2.1 [20, 21]. The cells were filtered based on their number of gene features and percentage of mitochondrial genes. The threshold used for gene features is between 2000 and 7500, and less than 10% mitochondrial gene expression. Genes to be used in analysis were expressed in at least 10 cells. LogNormalize function was used to normalize gene expression within each cell, by dividing the counts of each gene by the total counts in the cell, followed by a scale factor multiplication of 10,000 and then a natural log-transform of the result. Variable genes were identified using the FindVariableFeatures function and used to perform a principal component (PC) analysis using RunPCA. Within all the PCs, we used the top 20 PCs to do Uniform Manifold Approximation and Projection (UMAP) analysis. Clustering was carried out using the FindClusters function. Gene expression differences between the various cell clusters were carried out using FindAllMarkers function, filtering out genes expressed in less than 25% of cells in the compared clusters, and selecting genes with differential expression levels above 25%. Finally, clusters were identified by going through the list of differentially expressed genes and comparing the highly expressed genes with the published literature.

For the comparison with the HFO dataset [12], the dataset was retrieved from the GEO database (GEO Accession GSE150202). The raw data were downloaded using SRA-Toolkit and processed using cellranger 6.1.2. Similar preprocessing of the filtered matrices was carried out using R library *Seurat* version 3.2.1 [20, 21]**.** HFO dataset was combined with the CCO dataset using the integration functions FindIntegrationAnchors and IntegrateData. Downstream analysis was carried out in a similar manner.

### Video computational analysis

Video clips of the organoids were imaged using Nikon ECLIPSE Ti-S fluorescent microscope and recorded using an Andor Zyla 4.2 sCMOS. Images were extracted from each video clip at a frequency of 1/10^th^ second using ffmpeg [22], generating approximately 300 images from each video. The images were analysed using an image analysis pipeline implemented using Cell Profile 3.1.9 [23]. Organoid boundary and inner chamber regions were independently segmented from the images and used to compute the organoid size (outer area), the inner chamber area, the heart wall area and the heart wall area as a fraction of the organoid area (Additional file 6: Fig. S6). The organoid contraction peaks were computed in R by taking the first-order differential maximas of the organoid area with respect to time. The contraction peaks were then used to compute the individual organoid contraction rate and variations by taking the mean and standard deviation of the contraction intervals, respectively. Fractional shortening was determined by computing the percentage change of the heart wall thickness between the maximum relaxation of a diastole and the maximum contraction of a systole.

### Transfection and clonal isolation of hPSCs

hPSCs were dissociated using Accutase (Nacalai Tesque) at 37 °C for 3 min and 250K cells were seeded overnight on single wells of a 6-well plate. Plasmids were transfected into hPSCs seeded overnight on culture plates using Lipofectamine™ Stem transfection reagent (Invitrogen™). hPSCs were resuspended in phosphate-buffered saline (PBS) containing 0.5% foetal bovine serum (FBS), 1% bovine serum albumin (BSA), 1% Penicillin/Streptomycin (Gibco, U.S.A.) and 5 μM Y27632 (Miltenyi Biotec, Germany) and sorted into single wells using the LSR II (BD Biosciences, U.S.A).

GCaMP6s was stably knocked into each hPSC cell line using a Cas9 homology-directed repair protocol. *AAVS1* targeting gRNA was cloned into pSPCas9(BB)-2A-GFP (Addgene #48138; a gift from Feng Zhang). GCaMP6s donor plasmid was generated by inserting the GCaMP6s PCR amplicon from pGP-CMV-GCaMP6s (Addgene #40753; a gift from Douglas Kim & GENIE Project) into AAVS1-CAG-hrGFP (Addgene #52344; a gift from Su-Chun Zhang), replacing GFP. Two days after transfection, cells were first selected using 1ug/ml puromycin for two days and thereafter sorted for clonal isolation.

### Fluorescent imaging of intracellular calcium transient

Intracellular calcium kinetics was measured using H7-GCaMP6s-derived CCOs. Prior to imaging, fresh RPMI/B27 medium was added. Spontaneous calcium transients of control, 50 ng/ml, and 100 ng/ml EDN1-treated CCOs were imaged using Nikon ECLIPSE Ti-S fluorescent microscope and recorded using an Andor Zyla 4.2 sCMOS for 1 min. Calcium transients based on fluorescence intensity over time were computed into contraction peaks in RStudio. The contraction peaks were processed into the following variables: (1) calcium transient duration (seconds), (2) calcium transient peak intensity (Au), (3) depolarization speed (mean fluorescence decreased per second), and (4) repolarization duration (Repolarisation30_M, Repolarisation60_M, Repolarisation90_M) by taking the mean of the contraction repolarization duration of all peaks.

### Live organoid imaging

Live CCOs were placed on Nunc™ Lab-Tek™ II Chamber Slide™ System supplemented with CGM and imaged using Zeiss LSM780 Inverted Confocal Microscope with Environmental Chamber (10x/0.3 Plan-Neofluar objective lenses), 2 × Multialkali PMT + 2 × APD confocal detectors. Images were acquired using Zen (black) 2010. Image resolution was captured at 2048 × 2048 pixels. Live imaging was performed under humidified atmosphere of 5% CO_2_, 37 °C. Confocal images were captured at 2-h intervals (post-organoid generation) up to 24-h (post-organoid generation). Images shown in Fig. 2C were performed using a 20X objective lens multi-photon laser tuned to 940 nm, set to 20% laser power (Mai Tai, Spectra-Physics), with 5 × 5 (day 0) and 4 × 4 (day 1) tile scan. Organoids stained with Hoechst33342 (Miltenyi Biotech, Cat #130-111-569) and Calcein AM (Invitrogen, Cat #L3224) were imaged with a 10 × objective lens and 405 nm and 488 nm laser, set to 40% laser power with 5 × 5 tile scan (Fig. 2D).

### Statistical analysis

Statistical analyses were performed either using GraphPad Prism 8 or R. Quantitative PCR values between groups were compared using two-tailed Student’s *t* tests. Cardiac organoid parameters comparison between groups was performed using two-tailed Mann–Whitney *U*-test when sample size is small; otherwise, Welch’s *t* test was used. Comparison of calcium kinetics parameters derived from the fluorescent data was performed using two-tailed Student’s *t* tests.

## Results

### Enrichment of cardiovascular progenitor cells enable self-organization of derived cardiomyocytes into chambered cardiac organoids

Numerous organoid protocols published have demonstrated that progenitor-equivalents are necessary for the differentiation and self-organization in organoids [2, 24–26]. As such, we reasoned that the proliferative ISL1^+^ cardiovascular progenitor cells have the differentiation and organizational potential to generate the appropriate populations of cardiovascular lineages and enable the self-organization of CCOs. Previous studies have demonstrated that high level of canonical Wnt signals is an essential component in the microenvironment that facilitates the expansion of CVPs and subsequently the proper formation of the heart chambers by not allowing CVPs to undergo premature differentiation into cardiomyocytes in the pharyngeal mesoderm [27, 28]. Evidently, clonally expanded ISL1^+^ CVPs are cardiomyogenic and can give rise to the cell types of the cardiac lineage through Wnt modulation and generated structures resembling cardiac chambers in vivo [14]. Taken together, we decided to examine the role of CVPs as the key driver for chamber formation in CCOs.

**Fig. 1:**
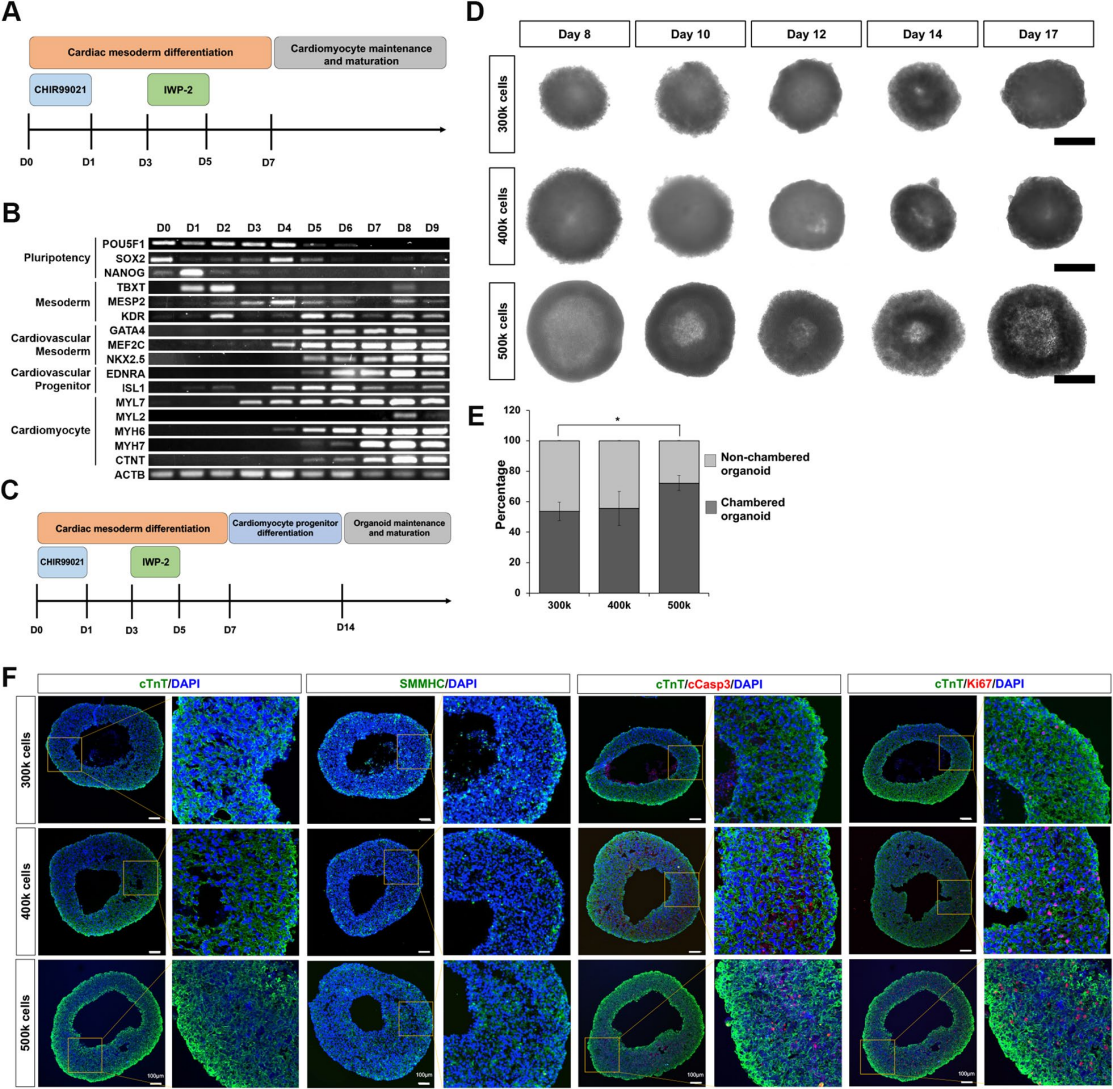
Generation and validation of human pluripotent stem cell-derived chambered cardiac organoids. **A** Schematic of the established hPSC-CM differentiation protocol. **B** RT-PCR tracking of relevant markers of cardiomyocyte differentiation from day 0 to day 9. **C** Schematic of the altered differentiation protocol to generate cardiac organoids. **D** Representative brightfield images of CCOs generated using 300k, 400k or 500k cells from day 8 to day 17. **E** Percentage of CCOs generated using 300k, 400k or 500k cells per organoid (*n* = 8 organoids per group from 3 experiments; mean ± s.d; *P = 0.0141; two-tailed Student’s *t* test). **F** Confocal immunofluorescence image of cardiomyocyte marker cTnT (green), smooth muscle cell marker SMMHC (green), proliferation marker Ki67 (red) and apoptosis marker cleaved Caspase-3 (red) in day 21 organoids generated from 300k, 400k or 500k cells. Cryosections were co-stained with DAPI (blue). Scale bar, 100 µm

To generate chambered cardiac organoids with an enriched population of CVPs, we tracked the lineage gene expression in hPSCs through 9 days of cardiomyocyte (CM) directed differentiation [17] and showed the progression of the hPSCs leaving pluripotency (D0 to D4), progressed towards the mesodermal lineage (D1 to D6), with a concomitant upregulation of cardiovascular mesodermal and progenitor genes (D4, peaking by D7), before CM markers were expressed (D5 onwards) (Fig. 1A, B). Thus, the generation of CCOs begins with the established 2D CM differentiation protocol, after which the cells were harvested and aggregated in the presence of Matrigel at Day 7. To promote the expansion of multipotent CVPs within the CCOs, cardiosphere growing media (CGM) was used to culture the organoids from D7 to D14 [14], followed by maturation in spinner flask culture using RPMI/B27 insulin media (Fig. 1C). The intrinsic formation of the chamber was tracked using brightfield imaging from D8 through D17, observed as a hollow core of a lighter shade present in the middle of the CCOs (Fig. 1D). A minimal cell number is required for chamber formation as organoids generated with 500k D7 cardiac differentiating cells was most robust in obtaining CCOs compared to 300k and 400k cells (Fig. 1E). Cryosection and immunocytochemistry of D21 CCOs showed cardiomyocyte (cTnT) and smooth muscle (SMMHC) organization around the chamber (Fig. 1F and Additional file 1: Fig. S1). Low levels of Ki67 + cells indicated the expected low proliferation rates of matured CMs (Fig. 1F). Of note, the intrinsically formed hollow chamber was not a consequence of apoptotic cells as the low levels of cleaved Caspase-3 staining indicated healthy, non-apoptotic CMs (Fig. 1F). Furthermore, as chamber formation was observed by Day 8, it is unlikely a consequence of apoptotic cells in the organoid core (Fig. 1D).

### Cellular threshold of CVPs essential for the formation of chambered cardiac organoids

**Fig. 2:**
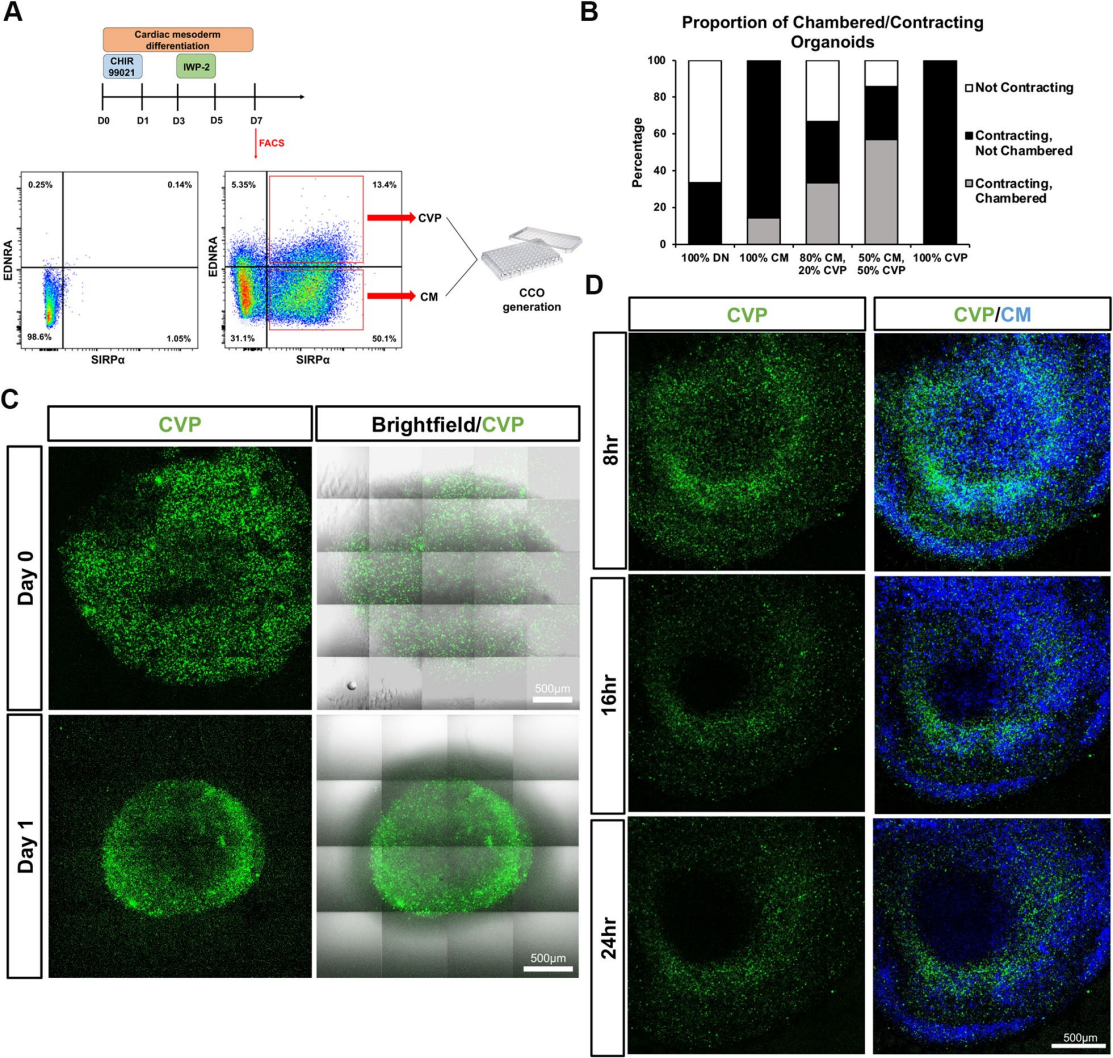
Cardiovascular progenitor cells play an essential role in the self-organization of a chambered cardiac organoid. **A** Mixed, control population stained with SIRPα and EDNRA fluorescent antibodies. SIRPα single-positive cells (bottom right quadrant) contain the cardiomyocyte population, while the SIRPα, EDNRA double-positive cells (top right quadrant) contain the cardiovascular progenitor population. **B** Percentages of contracting and chambered cardiac organoids generated using different compositions of cell populations. *DN* Double Negative Cells; *CM* SIRPA^+^ EDNRA^−^ Cardiomyocytes; *CVP* SIRPA^+^, EDNRA^+^ Cardiovascular Progenitor Cells (*n* = 6 per group). **C** Representative 2-Photon and brightfield images stitched from adjacent fields depicting CCOs generated using 50% CVPs and 50% CMs. CVPs were stained with Calcein AM (green). Scale bars, 500 µm. **D** Representative confocal images of CCOs generated using CVPs and CMs. CVPs were stained with Calcein AM (green) and Hoechst33342 (blue) and CMs were only stained with Hoechst33342. Scale bars, 500 µm

To further validate our hypothesis that CVPs are necessary for the intrinsic chamber formation, D7 CMs and CVPs were isolated using SIRPα [29] and EDNRA [14], respectively. As shown previously, EDNRA serves as a surface marker proxy to isolate ISL1^+^ CVPs [14]. SIRPα^−^/EDNRA^−^ double negative cells (DN), SIRPα^+^/EDNRA^−^ CMs and SIRPα^+^/EDNRA^+^ double positive CVPs were collected from the sort (Fig. 2A and Additional file 2: Fig. S2). Five compositions of organoids were generated [(1) 100% DN; (2) 100% CMs; (3) 80% CMs, 20% CVPs; (4) 50% CMs, 50% CVPs; (5) 100% CVPs], cultured to D21 and classified into three categories — not contracting, contracting without chamber formation, and contracting with chamber present (Fig. 2B). As expected, DN organoids of condition 1 either did not contract (67%) or contracted weakly (33%), while CM organoids of condition 2 were all contracting but most lacked chambers (86%) (Fig. 2B). Only in the conditions where both CVPs and CMs were present did the cardiac organoids contract and formed chambers, 33% and 57% of condition 3 and 4, respectively (Fig. 2B). Surprisingly, CVP organoids of condition 5 were all contracting with no chamber present, suggesting that not only are CVPs crucial in the formation of CCOs, there also exists an optimal balance between CVPs and CMs for sufficient cell-to-cell signalling for intrinsic chamber formation.

Furthermore, time-course confocal imaging was used to track the migration of CVPs over time after Matrigel encapsulation. CCOs with equal population of CVPs and CMs were generated, with CVPs stained prior with a green fluorophore, Calcein AM. Right after encapsulation (Day 0), CVPs were dispersed throughout the organoid, but by as early as Day 1 post-encapsulation, the CVPs have migrated and can be seen lining the inner chamber of the organoid while being noticeably missing from the exterior ring of the brightfield image (Fig. 2C). Hoechst staining further revealed that while the CVPs have migrated to the inner chamber, the CMs were still sparsely distributed across the entire CCO (Fig. 2D). The timing of CVP migration to the inner chamber is in agreement with the brightfield images which show the early stages of chamber formation in the Day 8 CCOs (Fig. 1D). These results highlight the importance of CVP presence and expansion in conjunction with cardiomyocytes to enable the generation of CCOs (Fig. 2E).

### Chambered cardiac organoids maintain the cardiovascular cell types found naturally in a human heart

**Fig. 3:**
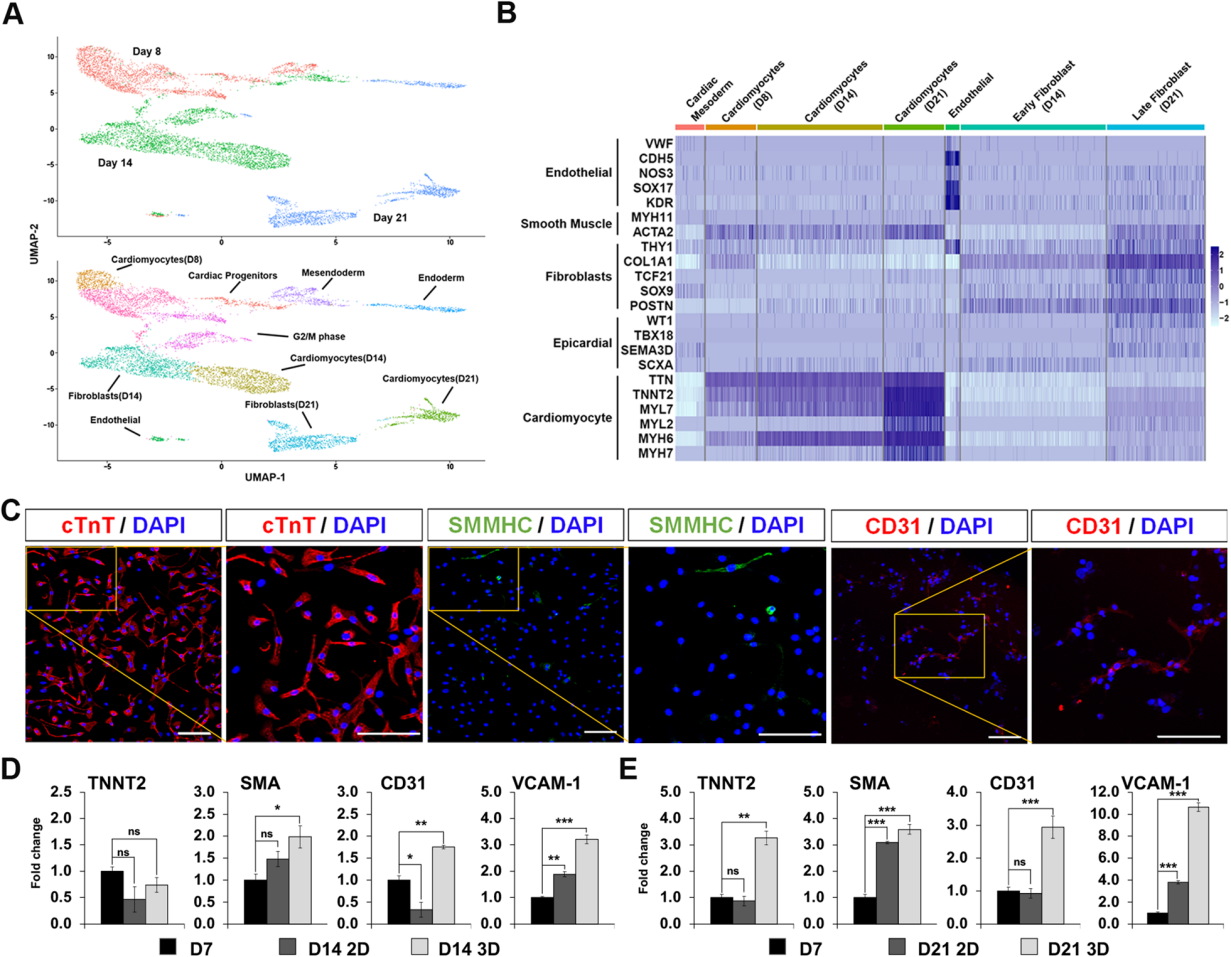
Chambered cardiac organoids are composed of cell types from the various cardiac lineages. **A** Uniform manifold approximation and projection (UMAP) of all cells, representing distinct identities of cells in day 8, day 14 and day 21 CCOs (top). UMAP revealed ten main cell clusters within the three time points (bottom). **B** Gene expression levels of the identified seven clusters related to the cardiac lineage are isolated in this heatmap. Each cluster identified is expressing their own marker genes. The singular endothelial cell cluster contains cells from all three timepoints of the organoids. **C** Representative confocal immunofluorescence image showing expression of cardiomyocyte, smooth muscle cell and endothelial cell lineage specific markers in the dissociated CCOs. Scale bars, 100 µm. **D–E** Quantitative RT-qPCR gene expression comparison of 2D cultured cardiomyocytes and 3D cardiac organoids of day 14 (**D**) and day 21 (**E**), normalized to day 7 cardiomyocytes. Genes shown represent markers of cardiomyocyte, smooth muscle and endothelial cell lineages (*n* = 3; mean ± s.e.m; housekeeping gene, *ACTB;* ***P < 0.001; **P < 0.01; *P < 0.05; two-tailed Student’s *t* test)

Transcriptome profiling using single-cell RNA sequencing showed that CCOs contain cell clusters present in a human heart and achieved increased expression of maturation-related genes across a time course from D8 (right after CCO formation) to D14 (after CVP expansion) followed by D21 (after 1 week of maturation) (Additional file 3: Fig. S3; D8: 2567 cells, D14: 3585 cells, D21: 1946 cells). Highly variable genes were used to identify the top 20 principal components for unsupervised clustering and Uniform Manifold and Projection (UMAP) non-linear dimensionality reduction, and 10 major cell clusters were assigned identities based on gene markers expressed (Fig. 3A, Additional file 13: Table S1) [30]. CM clusters of each time point were identified using CM markers (*TNNT2*^Hi^/*ACTN2*^Hi^/*TTN*^Hi^). A cardiac mesodermal cluster was identified only in D8 and D14 CCOs with cardiogenesis-related *SFRP2*, and other early mesodermal genes. An endothelial cluster containing cells from all 3 time points was recognized using endothelial markers (*CDH5*^Hi^/*SOX17*^Hi^/*KDR*^Hi^). An early cardiac fibroblast cluster and a late cardiac fibroblast cluster from D14 and D21, respectively, were classified based on increasing expressions of cardiac fibroblast markers (*THY1*/*TCF21*/*SOX9*/*POSTN*), with some cells in the cluster also expressing epicardial markers (*WT1*/*SEMA3D*/*TBX18*). A cluster of mesendodermal cells was identified in D8 and D14 CCOs (*TBX3*^Hi^/*FOXA2*^Hi^), that likely progressed to a cluster of definitive endodermal cells present only in D21 CCOs (*AFP*^+^/*TTR*^+^/*VIL1*^+^/*FOXA2*^+^). Clusters of dividing G_2_/M phase cells were only found in D8 and D14 CCOs, highly expressing cell cycling genes (*MKI67*^Hi^, *CDK1*^Hi^), likely related to the presence of dividing CVPs which are lost in D21 CCOs. Analysis of cardiovascular lineage clusters showed specific gene expression patterns which increase from earlier to later time points (Fig. 3B). This suggests CGM successfully expanded CVPs up to D14, before directed differentiation and maturation of the resident cardiovascular lineages by D21. Confirmation of the presence of the key cardiovascular lineages was demonstrated using immunofluorescence probing for the marker proteins—cTnT (CMs), SMMHC (smooth muscle cells) and CD31 (Endothelial cells) (Fig. 3C).

In comparison with classical 2D culture, quantitation of gene markers showed that smooth muscle (*SMA*) and vascular (*CD31*, *VCAM-1*) genes were upregulated in CCOs by D14 (Fig. 3D). By D21, a more pronounced increase in expression levels of CM (*TNNT2*), smooth muscle and vascular genes were observed (Fig. 3E). These findings indicate that the CCOs provide a microenvironment to maintain the various cardiovascular cell types. The presence of the various cardiovascular cell types is necessary for the CCOs to accurately model cardiovascular diseases.

To further understand the composition of the CMs in the CCOs, the markers indicative of the atrial/ventricular CMs, trabecular CMs as well as nodal cells in the day 21 CCO scRNA-seq results were assessed and depicted as feature plots (Additional file 4: Fig. S4). Cardiomyocytes expressing the atrial markers (*NR2F2*, *HEY1*, *PITX2*) form a small cluster at the bottom left of the UMAP plot (Additional file 4: Fig. S4A) [31–33], while the larger CM cluster at the top left of the UMAP plot indicates the larger presence of a majority of ventricular CMs expressing ventricular markers (*HEY2*, *IRX4*, *MYL2*) (Additional file 4: Fig. S4B) [31, 34, 35]. This result was expected as this CM generation protocol favours the generation of the ventricular lineage but does produce atrial CMs as well. Trabeculation markers are present and spread out across all the CMs on the left side of the UMAP plot. Overexpression of *CDH2* indicates the establishment of polarity necessary for trabecular morphogenesis, while expression of *CDKN1C*, *S1PR1* and *IRX3* markers confirm the presence of trabeculae CMs (Additional file 4: Fig. S4C) [36]. Lastly, the upregulation of *TBX3* at the small cluster at the bottom peak of the atrial CM cluster suggests the presence of nodal CMs as well (Additional file 4: Fig. S4D) [37].

### Chambered cardiac organoids promote maturation of resident cardiomyocytes

**Fig. 4:**
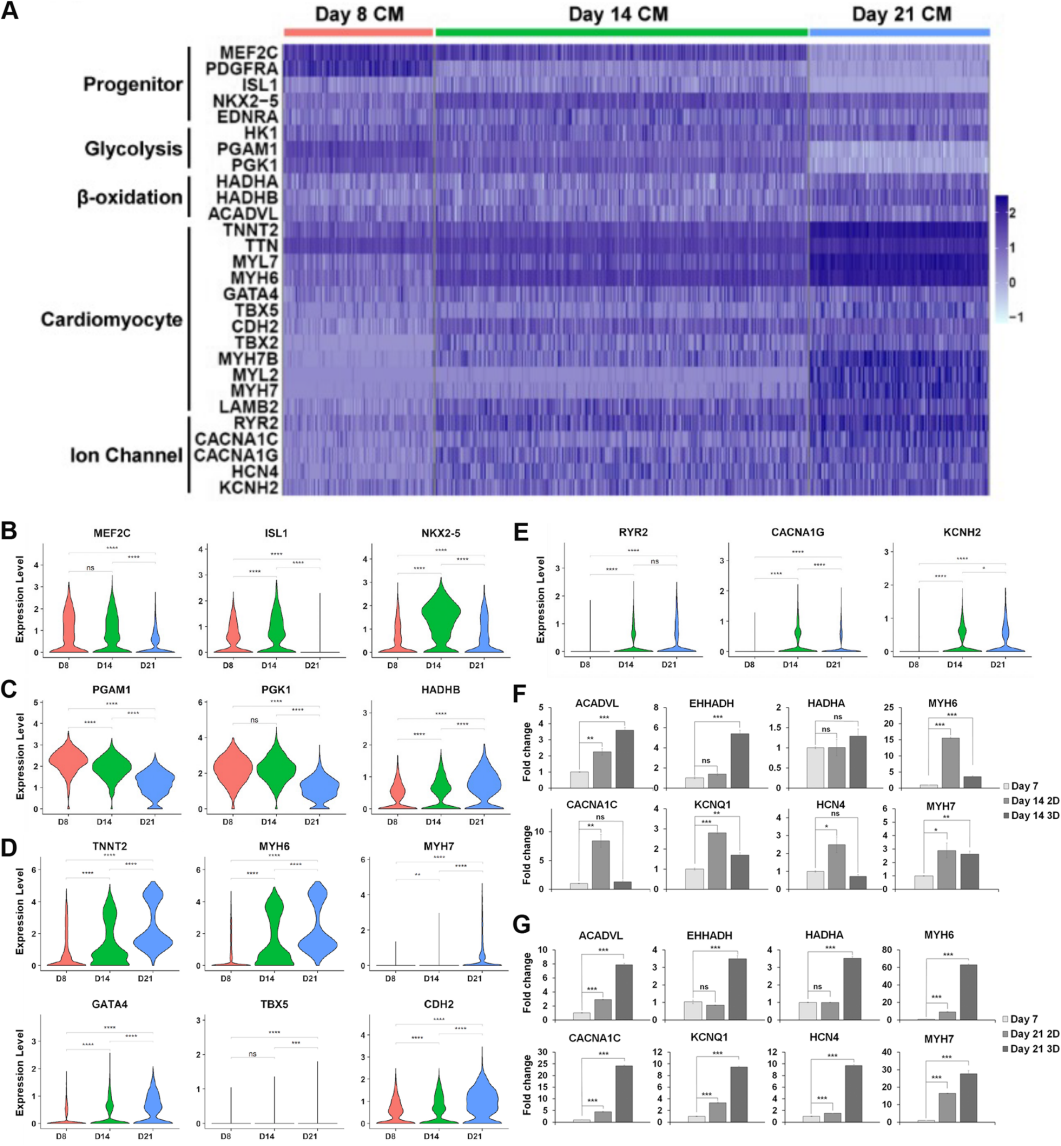
Chambered cardiac organoids enhance maturation of human embryonic stem cell-derived cardiomyocytes. **A** Heatmap depicting the increased maturation of cardiomyocyte clusters within the CCOs over the time points. **B–E** Violin plots highlighting key progenitor genes (**B**), Glycolytic *PGAM1*, *PGK1* and β-oxidation *HADHB* genes (**C**), cardiac genes (**D**) and ion channel gene (**E**) expression, corresponding to the changes in maturity of the cardiomyocyte clusters from day 8 (Red) to day 14 (Green) to day 21 (Blue). (****P < 0.0001; ***P < 0.001; **P < 0.01; *P < 0.05; ns *P* > 0.05; two-tailed Student’s *t* test). **F–G** Quantitative RT-qPCR gene expression comparison of 2D cultured cardiomyocytes and 3D cardiac organoids of day 14 (**F**) and day 21 (**G**), respectively, normalized to day 7 cardiomyocytes. Genes shown represent the pathways of β-oxidation, ion channel and cardiomyocyte markers (*n* = 3; mean ± s.e.m; housekeeping gene, *ACTB;* two-tailed Student’s *t* test)

Maturation of resident CMs is also crucial for disease modelling and can be measured by assessing the gene expression levels in the cardiomyocyte clusters corresponding to progenitors, glycolysis, β-oxidation, CM specificity, and ion channels (Fig. 4A). As expected, there is maintenance of CVP genes *MEF2C* and *ISL1*, and an increase in *NKX2.5* from CVP enrichment between D8 through D14 (Fig. 4B). Upon maintenance in maturation media, progenitor gene expression levels decreased, while *MEF2C* and *NKX2.5*, known to maintain CM identity, continued to be expressed [38]. On the contrary, *EDNRA* expression did not change over the time points, suggesting that while it marks the initial pool of CVPs together with ISL1 expression, EDNRA expression is still necessary for continued interactions with EDN1 and vascular organization. In addition, a metabolic switch from glycolysis to β-oxidation has been associated with CM maturation, and expectedly, glycolysis regulators *PGAM1* and *PGK1* were downregulated, while an upregulation of β-oxidation regulator *HADHB* was observed across the time points (Fig. 4C). Across the board, CM specificity markers increase across the time points, with late maturation marker, *MYH7* expressed only at D21 (Fig. 4D). A similar pattern was observed for ion channel genes with some markers only expressed in the later time points (Fig. 4E).

Quantitative gene expression comparison of maturation status with 2D cultured CMs showed expected results. By D14, while CCOs expressed increased β-oxidation markers *ACADVL* and *EHHADH*, CM specificity and ion channel markers are more significantly upregulated in 2D CMs, as the CCOs are being enriched for CVPs (Fig. 4F). By D21, all β-oxidation, CM and ion channel markers are highly upregulated in CCOs, highlighting marked improvements to maturation status compared to equivalent age 2D CMs (Fig. 4G). Thus, CCOs are able to achieve improved CM maturation over the same time course compared to 2D CMs.

To further demonstrate the relevance of CCOs for disease modelling, we compared the composition and maturity of our CCOs to the HFO dataset generated by Drakhlis et al*.* which modelled the development of the heart. As expected, the CCOs share a similar composition with the HFOs, containing mainly ventricular CMs, mesendodermal cells, endothelial cells as well as cardiac fibroblasts (Additional file 5: Fig. S5A and 5B, Additional file 14: Table S2). Most of the cell clusters determined from the combined dataset (Clusters 1 to 7) contain cells from both the CCO and HFO datasets (Additional file 5: Fig. S5C). Notably, Cluster 0 and 8 only contain cells from the CCOs, while the majority of cluster 1 is from the HFOs. Gene expression markers from the three clusters confirm that these are the CMs present in the organoids (Additional file 14: Table S2). Cluster 0 contains the younger, progenitor CMs, expressing markers such as *MEF2C*, which is only present in the early D8 and D14 CCOs due to the progenitor enrichment of the CGM media. Cluster 1 contains the bulk of the HFO CMs, expressing the cardiac markers such as *TNNT2* and *TTN*. The day 21 CCO CMs are contained within cluster 8, with higher expression of the same cardiac markers, while also expressing the more matured cardiac markers such as *MYL2* and *MYH7* (Additional file 5: Fig. S5D). These results support use of CCOs for disease modelling, being composed of the relevant cell types of enhanced maturity.

### Chambered cardiac organoids function as a disease model recapitulating cardiac hypertrophy

**Fig. 5:**
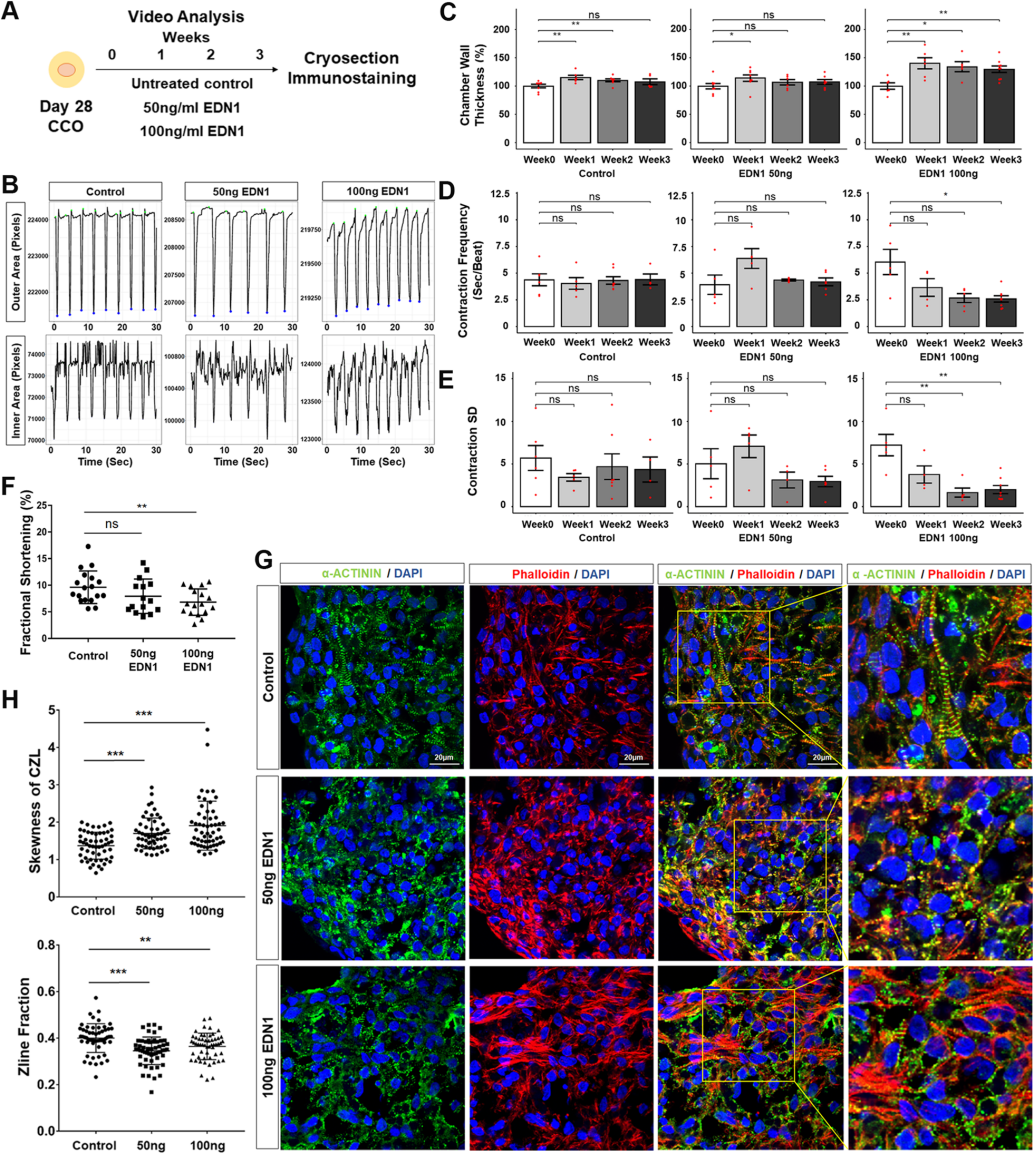
Cardiac hypertrophic phenotypes are recapitulated in chambered cardiac organoids treated with CH inducer EDN1. **A** Schematic diagram for cardiac hypertrophy modelling. **B** Outer (top) or inner (bottom) area against time plot of representative videos from a single CCO of each treatment condition. **C–E** Quantification of changes to chamber wall thickness normalized to Week 0 (**C**), contraction frequency (**D**), contraction standard deviation (**E**) across the time points**.** Each chart represents one treatment condition (Left: Control; Middle: 50 ng/ml EDN1; Right: 100 ng/ml EDN1). Each red dot represents one organoid sample analysed (*n* = 4–6 organoids per condition; mean ± s.e.m; Mann–Whitney U test). **F** Blind analysis of fractional shortening (%) in CCOs on the third week of treatment (Control *n* = 18, 50 ng/ml EDN1 *n* = 15, 100 ng/ml EDN1 *n* = 17; mean ± s.e.m; Welch’s *t* test). **G** Representative confocal immunofluorescence for z-line analysis of each condition at the end of Week 3. Scale bars, 20 µm. **H** Zline skewness (left) and Zline fraction (right) parameters are significantly affected in treated CCOs (*n* = 55 sections per group; mean ± s.e.m, Welch’s *t* test). In **(C**–**H)**, significance is presented as ***P < 0.001; **P < 0.01; *P < 0.05

Finally, to demonstrate functional utility of the CCO model, EDN1 was used as a cardiac hypertrophy (CH) inducer. Dysregulation of EDN1, a known vasoconstrictor, has been shown to cause CH and other cardiomyopathies [39–42]. To model CH, D28 CCOs were treated with two concentrations of EDN1 (50 and 100 ng/ml) and their contraction parameters assessed over 3 weeks of treatment (Fig. 5A). CCO contraction videos were processed to track the boundary of the entire organoid (outer area) and inner chamber (inner area) to compute chamber wall thickness (Fig. 5B, Additional file 6: Fig. S6, Additional file 7: Video S1, Additional file 8: Video S2, Additional file 9: Video 3). While there were minor increases in the chamber wall thickness in control and 50 ng/ml EDN1 treatment, only in the 100 ng/ml EDN1 treatment was it significantly sustained over three weeks (~ 30–40%) (Fig. 5C). The presence of the contracting heart wall and our ability to actively track the boundaries of the heart wall enabled to assessment of fractional shortening of the CCOs as a proxy of ejection fraction of an in vivo heart. The organoid contraction rate and rhythm were analysed to be used as a measure of arrhythmic tendencies. Contraction frequency increased significantly in the 100 ng/ml treatment group, doubling by week 3, while contraction variability decreased, correlating with the expected tachycardiac phenotype observed in clinical settings (Fig. 5D, E). Analysis of the CCO fractional shortening on the third week of treatment confirmed a significant ~ 30% decrease in the 100 ng/ml treatment group (Fig. 5F).

To functionally characterize the CCO contractile machinery, the sarcomeric architecture of CMs within the CCO was assessed using the computational structural assay ZlineDetection [19]. Earlier work demonstrated increased myofibrillar disarray in hPSC-CMs from CH patients [41]. Therefore, CCO cryosections stained with phalloidin and α-actinin were computationally assessed to compute the fraction of α-actinin composed of well-formed z-lines and the skewness of the continuous z-line (CZL) (Fig. 5G, H). Increasingly skewed CZL was observed as EDN1 treatment dosage increased and significantly reduced fraction of Z-lines were well formed in both EDN1 treated groups, both highlighting the myofibrillar disarray induced by EDN1 treatment (Fig. 5G). These functional findings are in line with other observations in CH models, wherein myofibrillar disarray results in the remodelling of contractile machinery and the thickening of the heart walls to counteract increased wall stresses [43].

**Fig. 6:**
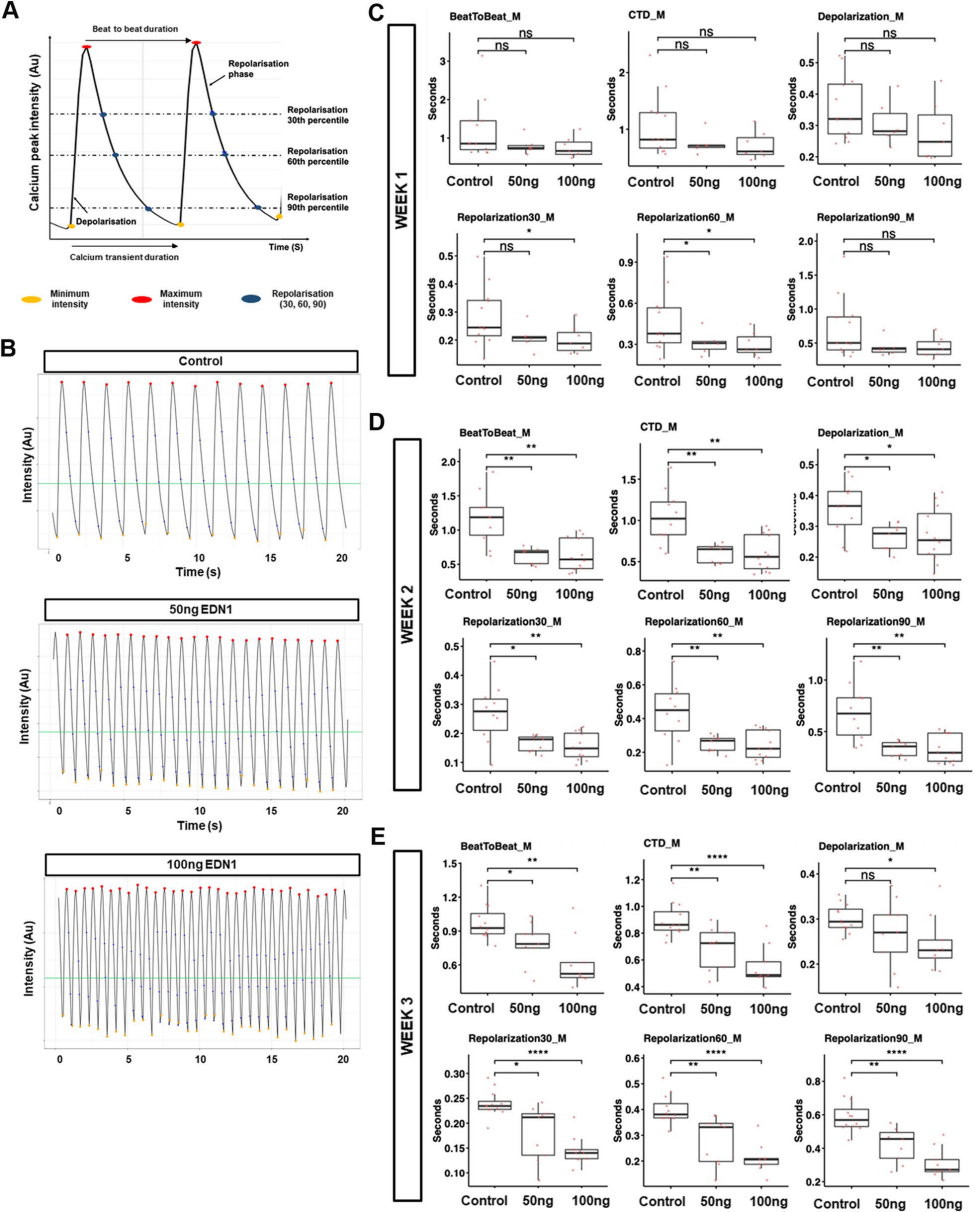
Altered calcium transients are recapitulated in chambered cardiac organoids treated with CH inducer EDN1. **A** Illustration depicting how the various calcium transient parameters were derived per single cardiomyocyte contraction rhythm. **B** Representative calcium transient profile in control, 50 ng/ml and 100 ng/ml EDN1-treated H7-GCaMP6s-derived CCOs. **C–E** Dot plot showing mean beat-to-beat duration (s), mean calcium transient duration (CTD) (s), mean depolarization duration (s), and repolarization duration (30^th^, 60^th^, 90^th^ per cent) across cells analysed for 1 week (**C**) 2 weeks (**D**) and 3 weeks (**E**) post-EDN1 treatment. Calcium transients per organoid was measured across 30 s. Each red dot represents one organoid sample analysed. *n* = 10 CCOs per group; mean ± s.e.m. Data information: Statistical analysis was performed using Students two-tailed *T* test. *P < 0.05, **P < 0.01, ***P < 0.001, ****P < 0.0001

Electrophysiological readouts are another necessary functional readout of a cardiovascular disease model. Thus, we generated CCOs from the H7 cell line expressing the GCaMP6s calcium reporter and tracked the calcium transient changes due to EDN1 treatment by fluorescent video recordings (Fig. 6A, B, Additional file 10: Video S4, Additional file 11: Video S5, Additional file 12: Video S6). Over the duration of the treatment, the electrophysiological phenotypes manifested in reduced beat-to-beat duration, reduced calcium transient duration and reduced depolarization duration. Notably, the effects of EDN1 treatment intensified over prolonged treatment and were also more severe in the higher dosage treatment of EDN1 (Fig. 6C–E). These results complemented the brightfield observations in Fig. 5C–F and confirmed that the tachyarrhythmic phenotype observed was a consequence of rapid depolarization occurring before complete repolarization, rather than an occurrence due to multiple ectopic foci. These findings are in agreement with in vivo hypertrophy models and clinical observations, which both point at hypertrophy leading to arrhythmogenic early or delayed after depolarizations [44, 45]. Thus, CCOs can be used as an electrophysiological analysis platform to distinguish the differing intensity of disease manifestations.

## Discussion

Chambered organoids represent a powerful platform to dissect genetic defects in vitro and potentially model translational research [12, 13]. Ravenscroft et al*.* and Giacomelli et al*.* demonstrated human heart organoids more closely recapitulated tissue physiology, showing contractile maturity, with matured electrophysiology and sarcomere structure [46, 47]. Herein, we utilized the multipotency of CVPs to establish self-organizing CCOs from hPSCs robustly. By modulating the Wnt pathway, the expansion of CVPs successfully demonstrated key developmental hallmarks to intrinsically specify, pattern and morph into the chambered 3D organoids. The mesodermal Wnt-Bmp signalling axis associated with the development of heart chambers enabled the inward migration and differentiation of CVPs lining the inner diameter of COOs [12]. These findings are consistent with the results based on HFOs recently reported by Hofbauer et al*.* and further illustrate the interplay between CVPs and Wnt signalling for chamber formation [12]. Importantly, we demonstrated the significance of CVPs in cardiac chamber formation by titrating the ratios of CVPs to CMs during CCO generation, thereby demonstrating an enriched population of CVPs was essential for chamber formation in vitro. Specifically, CVPs were found to be restricted towards a cardiac lineage while actively migrate to self-organize and line the inner chamber during CCO formation (Fig. 2E). Tapping unto the CVPs capacity to self-renew, demonstrated in our previous work, CCOs can be readily generated robustly to be used as a high-throughput platform for research [14].

Importantly, the current study also validated the utility of using CCOs for modelling cardiovascular diseases such as cardiac hypertrophy. Endothelin-1 (EDN1), known to induce oxidative stress in hiPSC-CMs, has been shown to promote post-translational modification in sarcomere and myofilament proteins, hence altering the actin–myosin protein interactions and causing myofibrillar disarray [48]. The disarray of the contractile apparatus as shown in Fig. 5G sets the stage for myocardial energy depletion triggering adaptive responses. Functional data from the CCO CH model strongly correlate with clinical observations of patients undergoing CH [49]. Existing literature suggests that CCOs exhibited these adaptive mechanisms by remodelling its contractile machinery, resulting in the thickening of the heart walls to counteract increased ventricular wall stresses, as well as increasing contraction rate to compensate for increased energy demands and reduced fractional shortening [43].

At present, there are other platforms that can assess parameters such as chamber wall thickening, ejection fraction and pressure generation [50–52]. Each of these alternative platforms suffer from their own set of drawbacks, but are primarily limited by the lack of self-organization and incomparability with actual human [53]. While existing 2D models demonstrate capabilities of measuring contraction rhythm and calcium transient dynamics in vitro, CCOs provide a platform to maintain and mature the various cardiovascular sub-types in 3D, mimicking the function of an in vivo heart and provided important output parameters such as heart wall thickness, fractional shortening, simultaneously. Other 3D disease models such as engineered heart tissues (EHTs) perform well in the measurement of parameters translating to contraction force. However, scaffolds are required to generate EHTs, which results in a platform less suitable for high-throughput generation and maintenance. In addition, the nature of EHTs typically containing predominantly cardiomyocytes prevents the study of cell–cell interaction between the various cell types of interest, which is not a limitation of CCOs. For example, the CVPs within the CCOs can be coaxed to either the cardiac fibroblast or endothelial lineage and maintained in suitable media and growth factors to study cell–cell interactions in cell-type specific CVDs such as cardiac fibrosis. Therefore, the CCO platform serves as a unique advancement of in vitro CVD modelling to complement current in vivo models, which also struggle to model cell–cell interactions due to the inherent complexity of animal hearts.

Recently, a machine learning algorithm was developed utilizing Ca^2+^ electrophysiological data generated using the GCaMP6s reporter to characterize arrhythmia using 2D monolayer CMs [54]. The machine learning framework requires large number of samples to generate reliable output which therefore led to the usage of hPSC-CMs to get the necessary sample sizes. CCOs can fill the niche as the 3D CM disease model, taking advantage the high-throughput nature of CCO generation and analysis compared to the other 3D CM disease models. Taken together, the high-throughput CCO platform used in conjunction with machine learning framework will aid in addressing the variability encountered when analysing contractile parameters and electrophysiology to model various forms of CVD.

## Conclusions

In this study, the necessity of CVPs and the role it plays in enabling the chamber formation in CCOs was demonstrated. These CCOs therefore represent a useful and significant advance for in vitro drug screening or disease modelling platforms to complement current in vivo models. The robustness of generating CCOs also hints at the possibility of generating chambered organoids of either atrial or ventricular identities by using sorted cardiomyocytes, such as atrial or ventricular cardiomyocytes for more specific modelling of cardiomyopathies.

## Supplementary Information

**Additional file 1: Fig. S1.** Confocal immunofluorescence images of cardiomyocyte and smooth muscle cell markers. Representative confocal images of cardiomyocyte marker cTnT (red) and smooth muscle cell marker SMMHC (green) expressed in day 21 CCOs. Cryosections were co-stained with DAPI (blue). Scale bar, 100 µm.

**Additional file 2: Fig. S2.** Sorting parameters used to obtain individual cell populations to generated chambered cardiac organoids of varying compositions. **A** Unstained, mixed control population of day 7 differentiated iPSC-cardiomyocytes. **B** Mixed, control population stained with SIRPα and EDNRA fluorescent antibodies. SIRPα single-positive cells (bottom right quadrant) contain the cardiomyocyte population, while the SIRPα, EDNRA double-positive cells (top right quadrant) contain the cardiovascular progenitor population.

**Additional file 3: Fig. S3.** Quality control filtering of the dataset to only include true cells of high quality using two metrics – Number of RNA features and levels of mitochondrial gene expression. **A** Unfiltered RNA gene detection levels across all single cell reads. **B** Unfiltered percentage of mitochondrial DNA detected within all single cell reads. **C** Filtered RNA gene detection levels across all single cell reads, keeping cells that express between 2000 – 7500 unique genes. **D** Filtered percentage of mitochondrial DNA detected within all single cell reads, keeping cells that have < 10% mitochondrial DNA.

**Additional file 4: Fig. S4.** UMAP feature plots depicting the markers of the various CM subtypes present in Day 21 CCOs. **A** NR2F2, HEY1 and PITX2 atrial marker expression. **B** HEY2, IRX4 and MYL2 ventricular marker expression. **C** CDH2, CDKN1C, S1PR1 and IRX3 trabecular marker expression. **D** TBX3 nodal marker expression.

**Additional file 5: Fig. S5.** Comparison of CCO scRNA-seq dataset with D13 HFO scRNA-seq dataset. **A** UMAP analysis of the CCO and HFO combined scRNA-seq dataset. Each single cell is coloured based on their sample origin. **B** Clustering based on UMAP determined 9 cell clusters. The younger CMs refer to clusters 0 and 1, while the matured CMs refer to cluster 8. **C** Plot showing the integration between the cells from the different origins into the various clusters. **D** Feature plot highlighting the expression of the various cardiac gene markers on the combined dataset.

**Additional file 6: Fig. S6.** Schematic depicting the pipeline of image segmentation. For every organoid video processed, individual frames were processed in accordance with the pipeline to obtain clear boundaries of the outer area and the inner area of the organoid. These boundaries were picked up by the in-house algorithm to segment the image into inner and outer chambers.

**Additional file 7: Video S1.** Representative brightfield video tracking the contractions of Week 3 untreated control chambered cardiac organoid.

**Additional file 8: Video S2.** Representative brightfield video tracking the contractions of Week 3 post 50 ng/ml EDN1 treatment chambered cardiac organoid.

**Additional file 9: Video S3.** Representative brightfield video tracking the contractions of Week 3 post 100 ng/ml EDN1 treatment chambered cardiac organoid.

**Additional file 10: Video S4.** Representative fluorescence imaging of Week 3 untreated control chambered cardiac organoid.

**Additional file 11: Video S5.** Representative fluorescence imaging of Week 3 post 50 ng/ml EDN1 treatment chambered cardiac organoid.

**Additional file 12: Video S6.** Representative fluorescence imaging of Week 3 post 100 ng/ml EDN1 treatment chambered cardiac organoid.

**Additional file 13: Table S1.** Table of top 100 differentially expressed genes upregulated between clusters identified from the scRNA-seq data across the three timepoints of organoids. Annotations retrieved from Ensembldb database.

**Additional file 14: Table S2.** Table of top 15 differentially expressed genes upregulated between clusters identified from the scRNA-seq data across the combined CCO and HFO samples. Annotations retrieved from Ensembldb database.

**Additional file 15: Table S3.** List of qPCR primers used in this study.

## Data Availability

The data discussed in this publication have been deposited in NCBI's Gene Expression Omnibus and are accessible through GEO Series accession GSE168464. The comparison dataset used can be accessed through the GEO Series accession GSE150202.

## Abbreviations

CCO: Chambered cardiac organoids
EDN1: Endothelin-1
hPSCs: Human pluripotent stem cells
hiPSC: Human-induced pluripotent stem cells
hiPSC-CMs: Human-induced pluripotent stem cell-derived cardiomyocytes
CM: Cardiomyocytes
CVP: Cardiovascular progenitors
CZL: Continuous z-line
scRNA-seq: Single-cell RNA sequencing
HFO: Heart-forming organoids
CH: Cardiac hypertrophy
EHT: Engineered heart tissues
CGM: Cardiosphere growing medium
