# Supplementary figures and images for: Robust generation of human-chambered cardiac organoids from pluripotent stem cells for improved modelling of cardiovascular diseases
Source: Stem Cell Res Ther. 2022 Dec 21;13:529. doi: 10.1186/s13287-022-03215-1 (PMC9773542; doi:10.1186/s13287-022-03215-1)

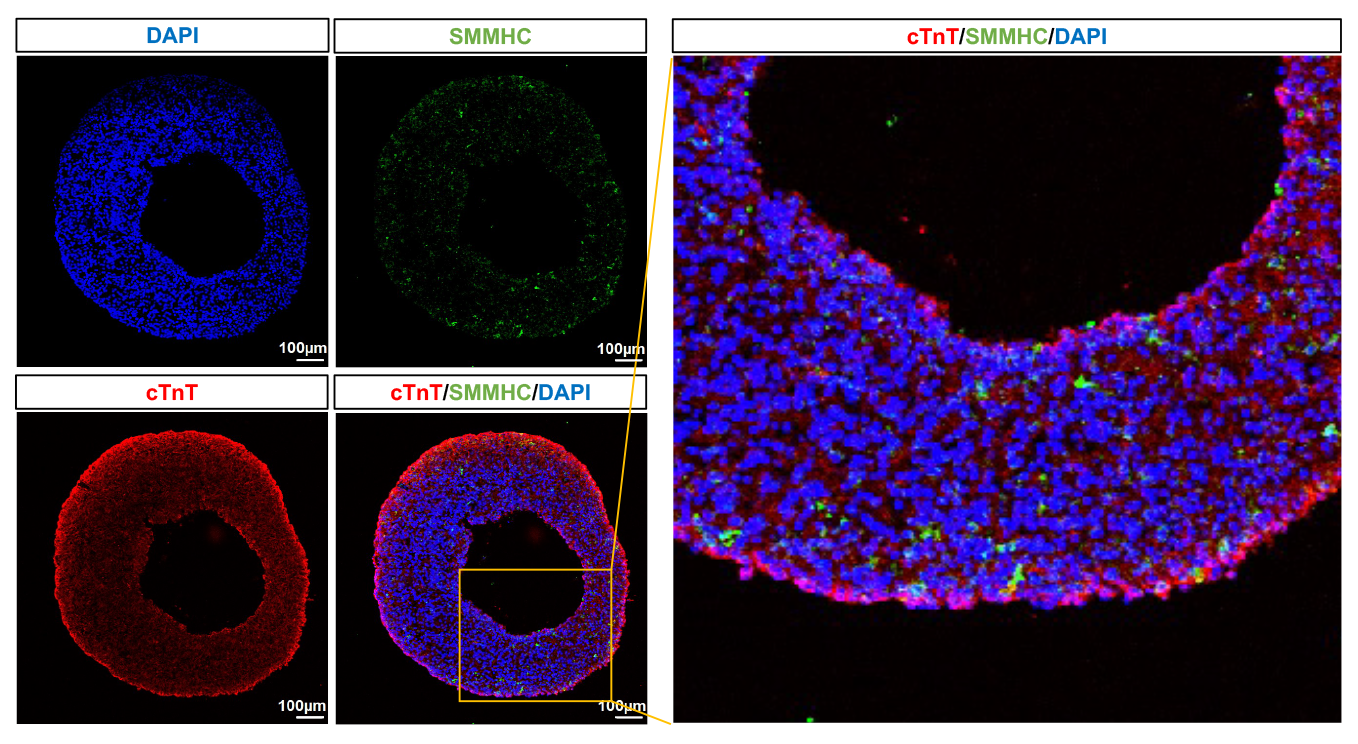

Supplement: Supplementary file 1 — Additional file 1: Fig. S1. Confocal immunofluorescence images of cardiomyocyte and smooth muscle cell markers. Representative confocal images of cardiomyocyte marker cTnT (red) and smooth muscle cell marker SMMHC (green) expressed in day 21 CCOs. Cryosections were co-stained with DAPI (blue). Scale bar, 100 µm. [file 13287_2022_3215_MOESM1_ESM.tif]

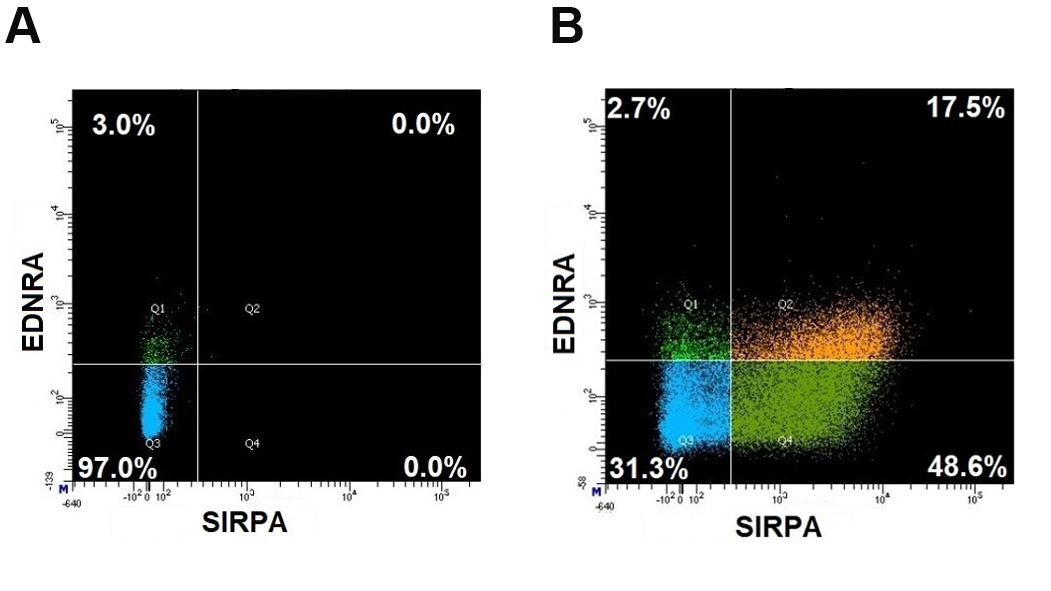

Supplement: Supplementary file 2 — Additional file 2: Fig. S2. Sorting parameters used to obtain individual cell populations to generated chambered cardiac organoids of varying compositions. A Unstained, mixed control population of day 7 differentiated iPSC-cardiomyocytes. B Mixed, control population stained with SIRPα and EDNRA fluorescent antibodies. SIRPα single-positive cells (bottom right quadrant) contain the cardiomyocyte population, while the SIRPα, EDNRA double-positive cells (top right quadrant) contain the cardiovascular progenitor population. [file 13287_2022_3215_MOESM2_ESM.tif]

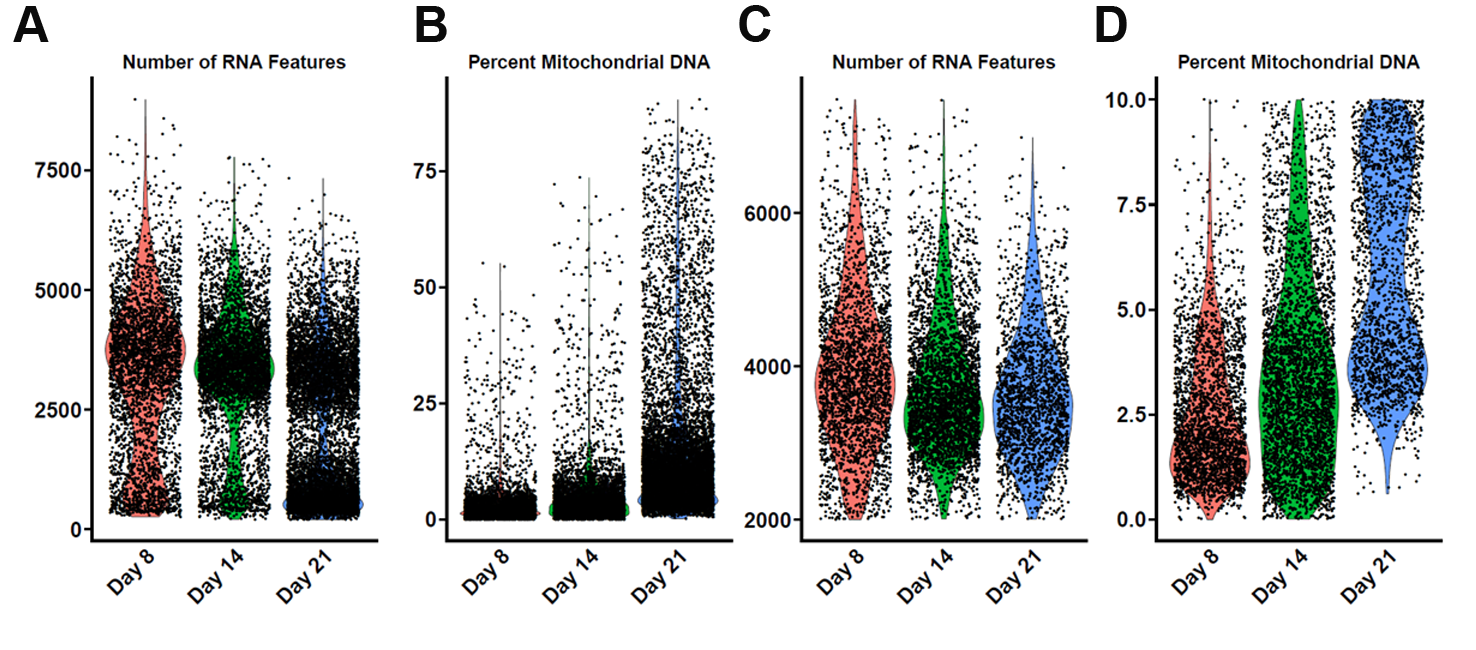

Supplement: Supplementary file 3 — Additional file 3: Fig. S3. Quality control filtering of the dataset to only include true cells of high quality using two metrics – Number of RNA features and levels of mitochondrial gene expression. A Unfiltered RNA gene detection levels across all single cell reads. B Unfiltered percentage of mitochondrial DNA detected within all single cell reads. C Filtered RNA gene detection levels across all single cell reads, keeping cells that express between 2000 – 7500 unique genes. D Filtered percentage of mitochondrial DNA detected within all single cell reads, keeping cells that have < 10% mitochondrial DNA. [file 13287_2022_3215_MOESM3_ESM.tif]

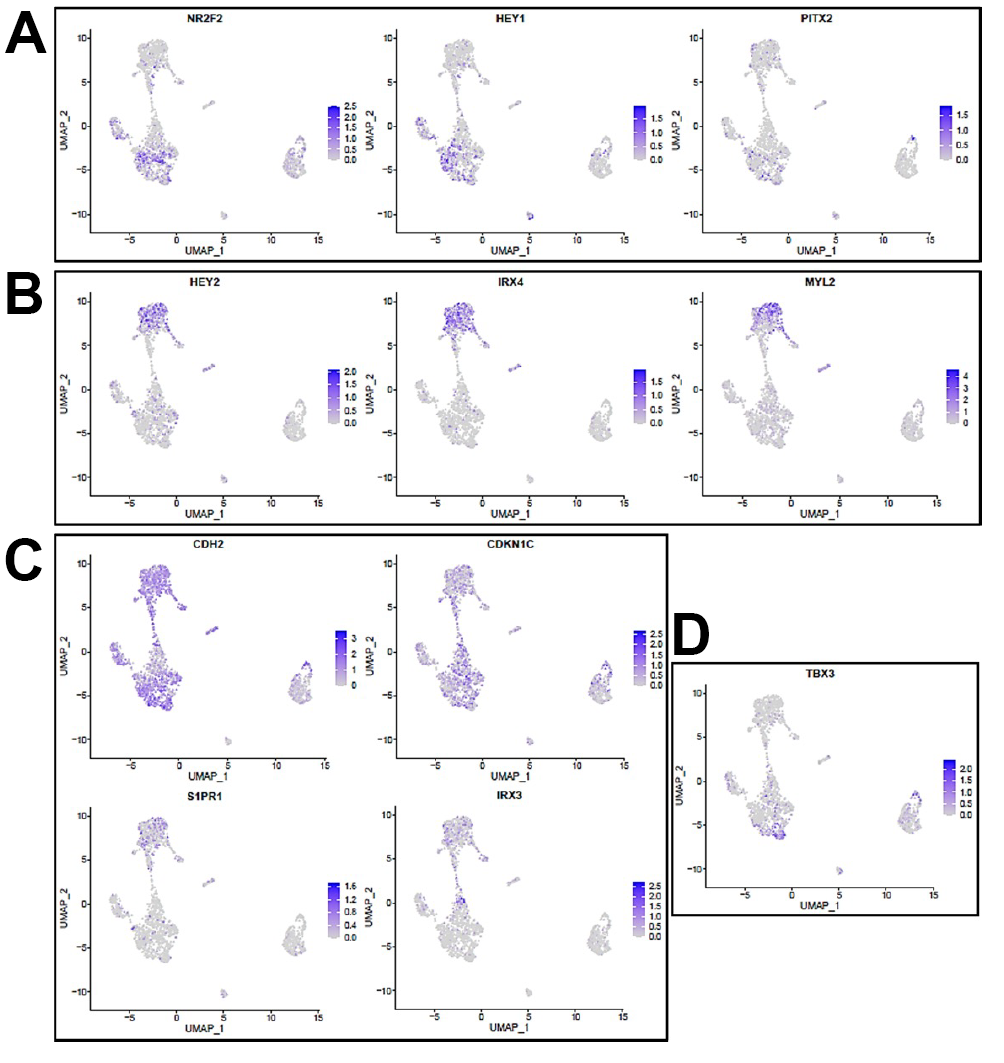

Supplement: Supplementary file 4 — Additional file 4: Fig. S4. UMAP feature plots depicting the markers of the various CM subtypes present in Day 21 CCOs. A NR2F2, HEY1 and PITX2 atrial marker expression. B HEY2, IRX4 and MYL2 ventricular marker expression. C CDH2, CDKN1C, S1PR1 and IRX3 trabecular marker expression. D TBX3 nodal marker expression. [file 13287_2022_3215_MOESM4_ESM.tif]

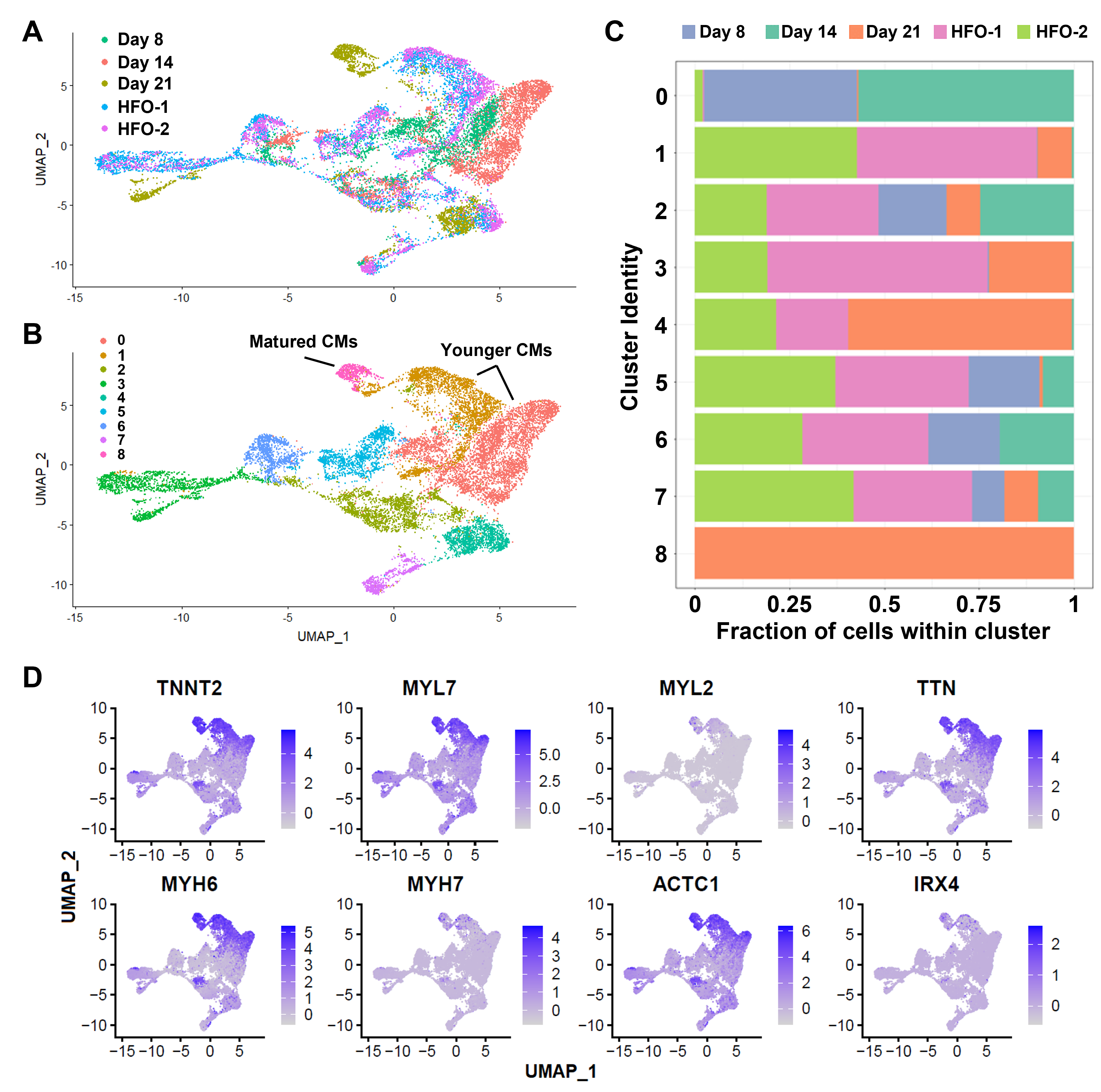

Supplement: Supplementary file 5 — Additional file 5: Fig. S5. Comparison of CCO scRNA-seq dataset with D13 HFO scRNA-seq dataset. A UMAP analysis of the CCO and HFO combined scRNA-seq dataset. Each single cell is coloured based on their sample origin. B Clustering based on UMAP determined 9 cell clusters. The younger CMs refer to clusters 0 and 1, while the matured CMs refer to cluster 8. C Plot showing the integration between the cells from the different origins into the various clusters. D Feature plot highlighting the expression of the various cardiac gene markers on the combined dataset. [file 13287_2022_3215_MOESM5_ESM.tif]

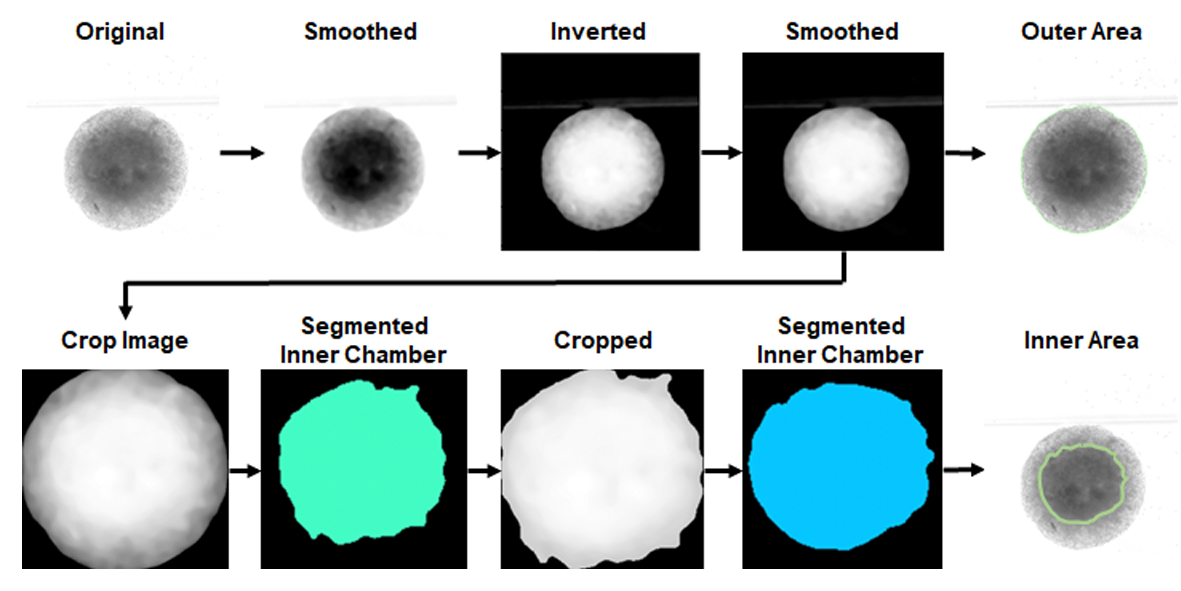

Supplement: Supplementary file 6 — Additional file 6: Fig. S6. Schematic depicting the pipeline of image segmentation. For every organoid video processed, individual frames were processed in accordance with the pipeline to obtain clear boundaries of the outer area and the inner area of the organoid. These boundaries were picked up by the in-house algorithm to segment the image into inner and outer chambers. [file 13287_2022_3215_MOESM6_ESM.tif]
